# STDP Installs in Winner-Take-All Circuits an Online Approximation to Hidden Markov Model Learning

**DOI:** 10.1371/journal.pcbi.1003511

**Published:** 2014-03-27

**Authors:** David Kappel, Bernhard Nessler, Wolfgang Maass

**Affiliations:** Institute for Theoretical Computer Science, Graz University of Technology, Graz, Austria; University of Cambridge, UK and Humboldt-Universität zu Berlin, Germany

## Abstract

In order to cross a street without being run over, we need to be able to extract very fast hidden causes of dynamically changing multi-modal sensory stimuli, and to predict their future evolution. We show here that a generic cortical microcircuit motif, pyramidal cells with lateral excitation and inhibition, provides the basis for this difficult but all-important information processing capability. This capability emerges in the presence of noise automatically through effects of STDP on connections between pyramidal cells in Winner-Take-All circuits with lateral excitation. In fact, one can show that these motifs endow cortical microcircuits with functional properties of a hidden Markov model, a generic model for solving such tasks through probabilistic inference. Whereas in engineering applications this model is adapted to specific tasks through offline learning, we show here that a major portion of the functionality of hidden Markov models arises already from online applications of STDP, without any supervision or rewards. We demonstrate the emergent computing capabilities of the model through several computer simulations. The full power of hidden Markov model learning can be attained through reward-gated STDP. This is due to the fact that these mechanisms enable a rejection sampling approximation to theoretically optimal learning. We investigate the possible performance gain that can be achieved with this more accurate learning method for an artificial grammar task.

## Author Summary

It has recently been shown that STDP installs in ensembles of pyramidal cells with lateral inhibition networks for Bayesian inference that are theoretically optimal for the case of stationary spike input patterns. We show here that if the experimentally found lateral excitatory connections between pyramidal cells are taken into account, theoretically optimal probabilistic models for the prediction of time-varying spike input patterns emerge through STDP. Furthermore a rigorous theoretical framework is established that explains the emergence of computational properties of this important motif of cortical microcircuits through learning. We show that the application of an idealized form of STDP approximates in this network motif a generic process for adapting a computational model to data: expectation-maximization. The versatility of computations carried out by these ensembles of pyramidal cells and the speed of the emergence of their computational properties through STDP is demonstrated through a variety of computer simulations. We show the ability of these networks to learn multiple input sequences through STDP and to reproduce the statistics of these inputs after learning.

## Introduction

**Figure 1:**
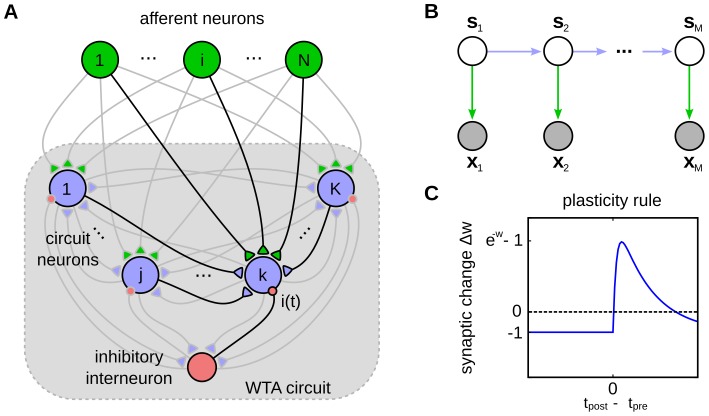
Illustration of the network model. (A) The structure of the network. It consists of 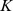 excitatory neurons (blue) that receive feedforward inputs (green synapses) and lateral excitatory all-to-all connections (blue synapses). Interneurons (red) install soft winner-take-all behavior by injecting a global inhibition to all neurons of the circuit in response to the network's spiking activity. (B) The Bayesian network representing the HMM over 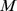 time steps. The prediction model (blue arrows) is implemented by the lateral synapses. It determines the evolution of the hidden states 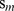 over time. The observation model (green arrows) is implemented by feedforward connections. The inference task for the HMM is to determine a sequence of hidden states 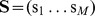 (white), given the afferent activity 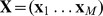 (gray). (C) The STDP window that is used to update the excitatory synapses. The synaptic weight change is plotted against the time difference between pre- and postsynaptic spike events.

An ubiquitous motif of cortical microcircuits is ensembles of pyramidal cells (in layers 2/3 and in layer 5) with lateral inhibition [1]–[3]. This network motif is called a *winner-take-all* (WTA) circuit, since inhibition induces competition between pyramidal neurons [4]. We investigate in this article which computational capabilities emerge in WTA circuits if one also takes into account the existence of lateral excitatory synaptic connections within such ensembles of pyramidal cells (Fig. 1A). This augmented architecture will be our default notion of a WTA circuit throughout this paper.

We show that this network motif endows cortical microcircuits with the capability to encode and process information in a highly dynamic environment. This dynamic environment of generic cortical mircocircuits results from quickly varying activity of neurons at the sensory periphery, caused for example by visual, auditory, and somatosensory stimuli impinging on a moving organism that actively probes the environment for salient information. Quickly changing sensory inputs are also caused by movements and communication acts of other organisms that need to be interpreted and predicted. Finally, a generic cortical microcircuit also receives massive inputs from other cortical areas. Experimental data with simultaneous recordings of many neurons suggest that these internal cortical codes are also highly dynamic, and often take the form of characteristic assembly sequences or trajectories of local network states [5]–[10]. We show in this article that WTA circuits have emergent coding and computing capabilities that are especially suited for this highly dynamic context of cortical microcircuits.

We show that spike-timing-dependent plasticity (STDP) [11], [12], applied on both the lateral excitatory synapses and synapses from afferent neurons, implements in these networks the capability to represent the underlying statistical structure of such spatiotemporal input patterns. This implies the challenge to solve two different learning tasks in parallel. First it is necessary to learn to recognize the salient high-dimensional patterns from the afferent neurons, which was already investigated in [13]. The second task consists in learning the temporal structure underlying the input spike sequences. We show that augmented WTA circuits are able to detect the sequential arrangements of the learned salient patterns. Synaptic plasticity for lateral excitatory connections provides the ability to discriminate even identical input patterns according to the temporal context in which they appear. The same STDP rule, that leads to the emergence of sparse codes for individual input patterns in the absence of lateral excitatory connections [13] now leads to the emergence of context specific neural codes and even predictions for temporal sequences of such patterns. The resulting neural codes are sparse with respect to the number of neurons that are tuned for a specific salient pattern and the temporal context in which it appears.

The basic principles of learning sequences of forced spike activations in general recurrent networks were studied in previous work [14], [15] and resulted in the finding that an otherwise local learning rule (like STDP) has to be enhanced by a global third factor which acts as an *importance weight*, in order to provide a – theoretically provable – approximation to temporal sequence learning. The possible role of such importance weights for probabilistic computations in spiking neural networks with lateral inhibition was already investigated earlier in [16].

In this article we establish a rigorous theoretical framework which reveals that each spike train generated by WTA circuits can be viewed as a sample from the state space of a *hidden Markov model* (HMM). The HMM has emerged in machine learning and engineering applications as a standard probabilistic model for detecting hidden regularities in sequential input patterns, and for learning to predict their continuation from initial segments [17]–[19]. The HMM is a generative model which relies on the assumption that the statistics of input patterns 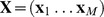 over 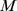 time steps is governed by a sequence of hidden states 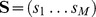, such that the 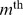 hidden state 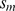 “explains” or generates the input pattern 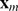. We show that the instantaneous state 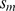 of the HMM is realized by the joint activity of all neurons of a WTA circuit, i.e. the spikes themselves and their resulting postsynaptic potentials. The stochastic dynamics of the WTA circuit implements a *forward sampler* that approximates exact HMM inference by propagating a single sample from the hidden state 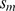 forward in time [19], [20].

We show analytically that a suitable STDP rule in the WTA circuit – notably the same rule on both the recurrent and the feedforward synaptic connections – realizes theoretically optimal parameter acquisition in terms of an online *expectation-maximization* (EM) algorithm [21], [22], for a certain pair 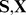 *if* the stochastic network dynamics describes the state sequence 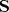 upon the input sequence 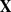. We further show that when the STDP rule is applied within the approximative forward sampling network dynamics of the WTA circuit, it instantiates a weak but well defined approximation of theoretically optimal HMM learning through EM. This is remarkable insofar as no additional mechanisms are needed for this approximation – it is automatically implemented through the stochastic dynamics of the WTA circuit, in combination with STDP. In this paper we focus on the analysis of this approximation scheme, its limits and its behavioral relevance.

We test this model in computer simulations that duplicate a number of experimental paradigms for evaluating emergent neural codes and behavioral performance in recognizing and predicting temporal sequences. We analyze evoked and spontaneous dynamics that emerges in our model network after learning an object sequence memory task as in the experiments of [23], [24]. We show that the pyramidal cells of a WTA circuit learn through STDP to encode the hidden states that underlie the input statistics in such tasks, which enables these cells to recognize and distinguish multiple pattern sequences and to autonomously predict their continuation from initial segments. Furthermore, we find neural assemblies emerging in neighboring interconnected WTA circuits that encode different abstract features underlying the task. The resulting neural codes resemble the highly heterogeneous codes found in the cortex [25]. Furthermore, neurons often learn to fire preferentially after specific predecessors, building up stereotypical neural trajectories within neural assemblies, that are also commonly observed in cortical activity [5]–[7], [26].

Our generative probabilistic perspective of synaptic plasticity in WTA circuits naturally leads to the question whether the proposed learning approximation is able to solve complex problems beyond simple sequence learning. Therefore we reanalyze data on artificial grammar learning experiments from cognitive science [27], where subjects were exposed to sequences of symbols generated by some hidden artificial grammar, and then had to judge whether subsequently presented unseen test sequences had been generated by the same grammar. We show that STDP learning in our WTA circuits is able to infer the underlying grammar model from a small number of training sequences.

The simple approximation by forward sampling, however, clearly limits the learning performance. We show that the full power of HMM-learning can be attained in a WTA circuit based on the *rejection sampling* principle [19], [20]. A binary factor is added to the STDP learning rule, that gates the expression of synaptic plasticity through a subsequent global modulatory signal. The improvement in accuracy of this more powerful learning method comes at the cost that every input sequence has to be repeated a number of times, until one generated state sequence is accepted. We show that a significant performance increase can be achieved already with a small number of repetitions. We demonstrate this for a simple and a more complex grammar learning task.

## Results

We first define the spiking neural network model for the winner-take-all (WTA) circuit considered throughout this paper. The architecture of the network is illustrated in Fig. 1A. It consists of stochastic spiking neurons, which receive excitatory input from an afferent population (green synapses) and from lateral excitatory connections (blue synapses) between neighboring pyramidal neurons. To clarify the distinction between these connections, we denote the synaptic efficacies of feedforward and lateral synapses by different weight matrices 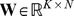 and 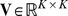, respectively, where 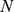 denotes the number of afferent neurons and 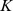 the size of the circuit (i.e., the number of pyramidal cells in the circuit). In addition, all neurons within the WTA circuit project to interneurons and in turn all receive the same common inhibition 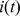. Thus the membrane potential of neuron 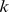 at time 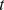 is given by
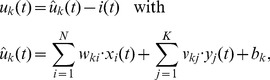
(1)

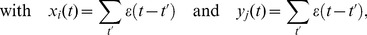
(2)where 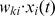 and 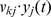 denote the time courses of the excitatory postsynaptic potentials (EPSP) under the feedforward and lateral synapses, where 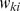 and 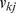 are the elements of 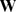 and 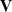 respectively, and 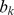 is a parameter that controls the excitability of the neuron. The two sums in (1) describe the time courses of the membrane potential in response to synaptic inputs from feedforward and lateral synapses. In equation (2) we used the assumption of additive EPSPs, where 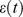 denotes a kernel function that determines the time course of an EPSP [28]. The sums run over all spike times of the presynaptic neuron. For the theoretical analysis we used a single exponential decay for the sake of simplicity, throughout the simulations we used double exponential kernels, if not stated otherwise. Our theoretical model can be further extended to other EPSP shapes (see the Methods section for details).

As proposed in [29], we employ an exponential dependence between the membrane potential and the firing probability. Therefore the instantaneous rate of neuron 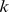 is given by 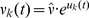, where 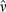 is a constant that scales the firing rate. The inhibitory feedback loop 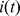 in equation (1), that depresses the membrane potentials whenever the network activity rises, has a normalizing effect on the circuit-wide output rate. Although, each neuron 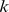 generates spikes according to an individual Poisson process, this inhibition couples the neural activities and thereby installs the required competition between all cells in the circuit. We model the effect of this inhibition in an abstract way, where we assume, that all WTA neurons receive the same inhibitory signal 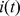 such that the overall spiking rate of the WTA circuit stays approximately constant. Ideal WTA behavior is attained if the network rate is normalized to the same value at any point in time, i.e. 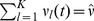. Using this, we find the circuit dynamics to be determined by
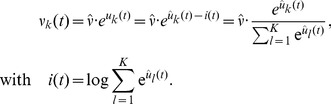
(3)This ideal WTA circuit realizes a soft-max or soft WTA function, granting the highest firing rate to the neuron with the highest membrane potential, but still allowing all other neurons to fire with non-zero probability.

### Recapitulation of hidden Markov model theory

In this section we briefly summarize the relevant concepts for deriving our theoretical results. An exhaustive discussion on hidden Markov model theory can be found in [17]–[19]. Throughout the paper, to keep the notation uncluttered we use the common short-hand notation 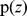 to denote 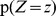, i.e. the probability that the random variable 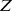 takes on the value 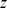. If it is not clear from the context, we will use the notation 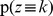 to remind the reader of the underlying random variable, that is only implicitly defined.

The HMM is a generative model for input pattern sequences over 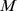 time steps 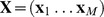 (the input patterns are traditionally called observations in the context of HMMs). It relies on the assumption that a sequence of hidden states 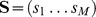 and a set of parameters 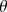 exist, which govern the statistics of 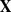. This assumption allows to write the joint distribution of 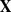 and 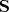 as
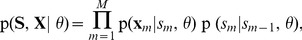
(4)where we suppress an explicit representation of the initial state 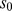, for the sake of brevity. The joint distribution (4) factorizes in each time step into the *observation model* 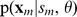 and the state transition or *prediction model* 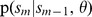 [19]. This independence property is illustrated by the Bayesian network for a HMM in Fig. 1B.

The HMM is a generative model and therefore we can recover the distribution over input patterns by marginalizing out the hidden state sequences 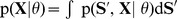. Learning in this model means to adapt the model parameters 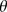 such that this marginal distribution 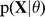 comes as close as possible to the empirical distribution 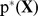 of the observable input sequences. A generic method for learning in generative models with hidden variables is the *expectation-maximization* (EM) algorithm [30], and its application to HMMs is known as the Baum-Welch algorithm [31]. This algorithm consists of iterating two steps, the *E-step* and the *M-step*, where the model parameters 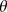 are adjusted at each M-step (for the updated posterior generated at the preceding E-step). A remarkable feature of the algorithm is that the fitting of the model to the data is guaranteed to improve at each M-step of this iterative process. Whereas the classical EM algorithm is restricted to offline learning (where all training data are available right at the beginning), there exist also stochastic online versions of EM learning.

In its stochastic online variant [21], [22] the E-step consists of generating one sample 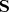 from the *posterior distribution* 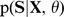, given one currently observed input sequence 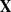. Given these sampled values for 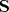, the subsequent M-step adapts the model parameters 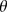 such that the probability 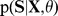 increases. The adaptation is confined to acquiring the conditional probabilities that govern the observation and the prediction model.

It would be also desirable to realize the inference and sampling of one such posterior sample sequence 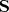 in a fully online processing, i.e. generating each state 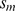 in parallel to the arrival of the corresponding input pattern 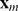. Yet this seems to be impossible as the probabilistic model according to (4) implies a statistical dependence between any 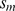 and the whole future observation sequence 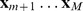. However, it is well known that the inference of 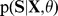 can be approximated by a so-called *forward sampling* process [19], [20], where every single time step 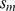 of the sequence 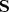 is sampled online, based solely on the knowledge of the observations 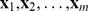 received so far, rather than the observation of the complete sequence 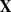. Hence sampling the sequence 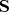 is approximated by propagating a single sample from the HMM state space forward in time.

### Forward sampling in WTA circuits

In this section we show that the dynamics of the network realizes a forward sampler for the HMM. We make use of the fact that equations (1), (2) and (3) realize a Markov process, in the sense that future network dynamics is independent from the past, given the current network state (for a suitable notion of network state). This property holds true for most reasonable choices of EPSP kernels. For the sake of brevity we focus in the theoretical analysis on the simple case of a single exponential decay with time constant 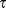.

We seek a description of the continuous-time network dynamics in response to afferent spike trains over a time span of length 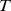 that can be mapped to the state space of a corresponding HMM with discrete time steps. Although the network works in continuous time, its dynamics can be fully described taking only those points in time into account, where one of the neurons in the recurrent circuit produces a spike. This allows to directly link spike trains generated by the network to a sequence of samples from the state space of a corresponding HMM.

Let the 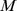 spike times produced during this time window be given by 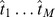. The neuron dynamics are determined by the membrane time courses (2). For convenience let us introduce the notation 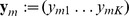, with 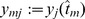 and by analogy 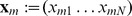, with 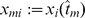.

Due to the exponentially decaying EPSPs the synaptic activation 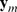 at time 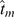 is fully defined by the synaptic activation 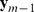 at the time of the previous spike 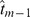, and the identity of the neuron that spiked in that previous time step, which we denote by a discrete variable 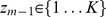. We thus conclude that the sequence of tuples 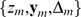 (with 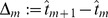) fulfills the Markov condition, i.e. the conditional independence 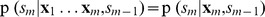 and thus fully represents the continuous dynamics of the network (see Methods). We call 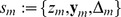 the *network state*. The corresponding HMM forward sampler follows a simple update scheme that samples a new state 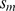 given the current observation 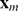 and the previous state 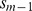. This dynamic is equivalent to the WTA network model.

This state representation allows us to update the network dynamics online, jumping from one spike time 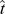 to the next. Using this property, we find that the dynamics of the network realizes a probability distribution over state sequences 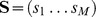, given an afferent sequence 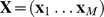, which can be written as
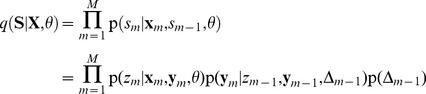
(5)where 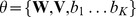 is the set of network parameters. The factorization and independence properties in (5) are induced by the state representation and the circuit dynamics. We assume here that the lateral inhibition within the WTA circuit ensures that the output rate of the whole circuit is normalized, i.e. 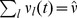 at all times 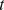. This allows to introduce the distribution over the inter-spike-time intervals 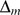 independent from 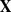 (see Methods for details). Note, that 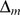 determines the interval between spikes of *all* circuit neurons, realized by a *homogeneous* Poisson process with a constant rate 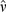. The second term in the second line of (5) determines the course of the membrane potential, i.e. it assures that 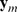 follows the membrane dynamics. Since the EPSP kernels are deterministic functions this distribution has a single mass point, where (2) is satisfied. The first factor in the second line of (5) is given by the probability of each individual neuron to spike. This probability depends on the membrane potential (1), which in turn is determined by 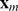, 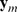 and the network parameters 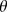. Given that the circuit spikes at time 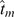, the firing probability of neuron 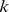 can be expressed as a conditional distribution 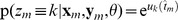. The lateral inhibition in (1) ensures that this probability distribution is correctly normalized. Therefore, the winner neuron 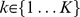 is drawn from a multinomial distribution at each spike time.

For the given architecture the functional parts of the network can be related directly to hidden Markov model dynamics. In the Methods section we show in detail that by rewriting 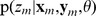 the membrane potential (1) can be decomposed into three functional parts
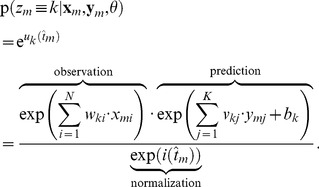
(6)The lateral excitatory connections predict a prior belief about the current network activity and the feedforward synapses match this prediction against the afferent input. The inhibition 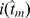 implements the normalization that is required to make (6) a valid multinomial distribution. The functional parts of the membrane potential can be directly linked to the prediction and observation models of a HMM, where the network state is equivalent to the hidden state of this HMM. The WTA circuit realizes a forward-sampler for this HMM, which approximates sampling from the posterior distribution 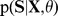 in an online fashion [20]. Its sampling is carried out step by step, i.e. it generates through each spike a new sample from the network state space, taking only the previous time step sample into account. Furthermore this forward sampling requires no additional computational organization, but is achieved by the inherent dynamics of the stochastically firing WTA circuit.

### STDP instantiates a stochastic approximation to EM parameter learning

Formulating the network dynamics in terms of a probabilistic model is beneficial for two reasons: First, it gives rise to a better understanding of the network dynamics by relating it to samples from the HMM state space. Second, the underlying model allows us to derive parameter estimation algorithms and to compare them with biological mechanisms for synaptic plasticity. For the HMM, this approach results in an instantiation of the EM algorithm [19], [30] in a network of spiking neurons (stochastic WTA circuit). In the Methods section we derive this algorithm for the WTA circuit and show that the M-step evaluates to weight updates that need to be applied whenever neuron 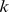 emits a spike at time 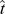, according to
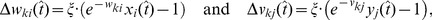
(7)where 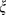 is a positive constant that controls the learning rate. Note that the update rules for the feedforward and the recurrent connections are identical, and thus all excitatory synapses in the network are handled uniformly. These plasticity rules (7) are equivalent to the updates that previously emerged as theoretically optimal synaptic weight changes, for learning to recognize repeating high-dimensional patterns in spike trains from afferent neurons, in related studies [13], [32], [33]. The update rules consist of two parts: A Hebbian long-term potentiating (LTP) part that depends on presynaptic activity and a constant depression term. The dependence on the EPSP time courses (2) makes the first part implicitly dependent on the history of presynaptic spikes. The STDP window is shown in Fig. 1C for 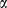-shaped EPSPs. Potentiation is triggered when the postsynaptic neuron fires after the presynaptic neuron. This term is commonly found in synaptic plasticity measured in biological neurons, and for common EPSP windows it closely resembles the shape of the pre-before-post part of standard forms of STDP [11], [12]. The dependence on the current value of the synaptic weight has a local stabilizing effect on the synapse. The depressing part of the update rule is triggered whenever the postsynaptic neuron fires independent of presynaptic activity. It contrasts LTP and assures that the synaptic weights stay globally in a bounded regime. It is shown in Fig. 4 of [13] that the simple rule (7) reproduces the standard form of STDP curves when it is applied with an intermediate pairing rate.

While these M-step updates emerge as exact solutions for the underlying HMM, the WTA circuit implements an approximation of the *E-step*, using forward sampling from the distribution in equation (5). In the following experiments we will first focus on this simple approximation, and analyze what computational function emerges in the network using the STDP updates (7) without any third signal related to reward or a “teacher”. In the last part of the Results section we will introduce a possible implementation of a refined approximation, and assess the advantages and disadvantages of this method.

### Learning to predict spike sequences through STDP

In this section we show through computer simulations that our WTA circuits learn to encode the hidden state that underlies the input statistics via the STDP rule (7). We demonstrate this for a simple sequence memory task and analyze in detail how the hidden state underlying this task is represented in the network. The experimental paradigm reproduces the structure of object sequence memory tasks, where monkeys had to memorize a sequence of movements and reproduce it after a delay period [23], [24], [34], [35]. The task consisted of three phases: An initial cue phase, a delay phase and a recall phase. Each phase is characterized by a different input sequence, where the cue sequence defines the identity of the recall sequence. We used four cue/recall pairs in this experiment.

**Figure 2:**
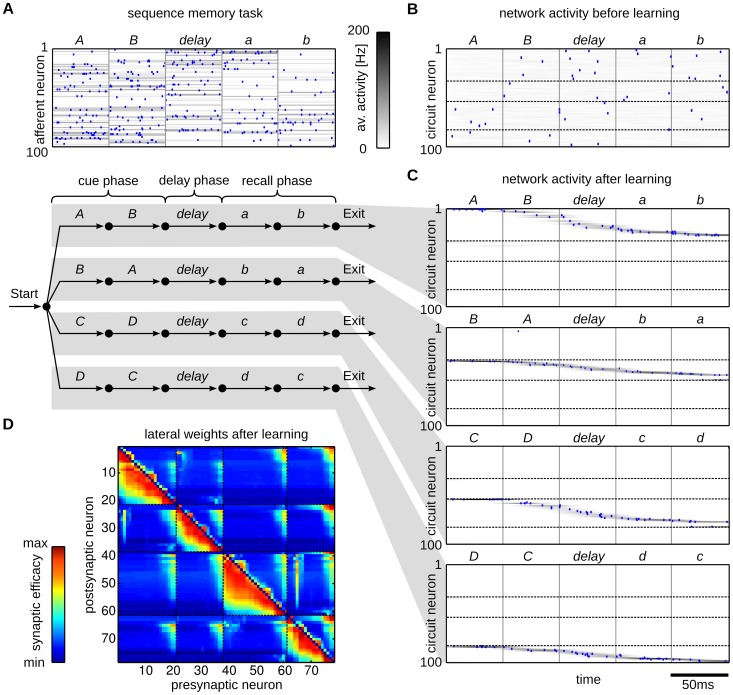
Emergence of working memory encoded in neural assemblies through weak HMM learning in a WTA circuit through STDP. (A) Illustration of the input encoding for sequence *AB-delay-ab*. The upper plot shows one example input spike train (blue dots) plotted on top of the mean firing rate (100 out of 200 afferent neurons shown). The lower panel shows the finite state grammar graph that represents the simple working memory task. The graph can be used to generate symbol sequences by following any path from *Start* to *Exit*. In the first state (*Start*) a random decision is made, which of the four paths to take. This decision determines all arcs that are passed throughout the sequence. On each arc that is passed the symbol next to the arc is emitted (and provided as input to the WTA circuit in the form of some 200-dimensional rate pattern). (B,C) Evoked activity of the WTA circuit for one example input sequence before learning (B) and for each of the four sequences after learning (C). The network activity is averaged and smoothed over 100 trial runs (gray traces), the blue dots show the spiking activity for one trial run. The input sequences are labeled by their pattern symbols on top of each plot. The neurons are sorted by the time of their highest average activity over all four sequences, after learning. For each sequence a different assembly of neurons becomes active in the WTA circuit. Dotted black lines indicate the boundaries between assemblies. Since the 4 assemblies that emerged have virtually no overlap, the WTA circuit has recovered the structure of the hidden states that underlie the task. (D) The lateral weights 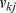 that emerged through STDP. The neurons are sorted using the same sorting algorithm as in (B,C). The black dotted lines correspond to assembly boundaries, neurons that fired on average less than one spike per sequence are not shown. Each neuron has learned to fire after a distinct set of predecessors, which reflects the sequential order of assembly firing. The stochastic switches between sequences are represented by enhanced weights between neurons active at the sequence onsets.

The structure of this task is illustrated in Fig. 2A. The graph represents a finite state grammar that can be used to generate symbol sequences by following a path from *Start* to *Exit*. In this first illustrative example the only stochastic decision is made at the beginning, randomly choosing one of the four cue phases with equal probabilities while the rest of the sequence is deterministic. On each arc that is passed, the symbol next to the arc is generated, e.g. *AB-delay-ab* is one possible symbolic sequence. Note that all symbols can appear in different temporal contexts, e.g. *A* appears in sequence *AB-delay-ab* and in *BA-delay-ba*. The *delay* symbol is completely unspecific since it appears in all four possible sequences. Therefore this task does not fulfill the Markov condition with respect to the input symbols, e.g. knowing that the current symbol is *delay* does not identify the next one as it might be any of *a,b,c,d*. Only additional knowledge about the temporal context of the symbol allows to uniquely identify the continuation of the sequence.

This additional knowledge can be represented in a hidden state that encodes the required information, which renders this task a simple example of a HMM. The hidden states of this HMM have to encode the input patterns and the temporal context in which they appear in order to maintain the Markov property throughout the sequences, e.g. a distinct state 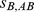 encodes pattern *B* when it appears in sequence *AB-delay-ab*. The temporal structure of the hidden state can be related to the finite state grammar in Fig. 2A. The arcs of the grammar directly correspond to the hidden states, i.e. given knowledge about the currently visited arc allows us to complete the sequence. The symbols next to the arcs define the observation model, i.e. the most likely symbol throughout each state. In this simple symbolic HMM the observation model is in fact deterministic, since exactly one symbol is allowed in each state.

In the neural implementation of this task, the symbolic sequences are presented to the WTA circuit encoded by afferent spike trains. Every symbol *A,B,C,D,a,b,c,d,delay* is represented by a rate pattern with fixed length of 50 ms, during which each afferent neuron emits spikes with a symbol-specific, fixed Poisson rate (see Methods). One example input spike train encoding the symbolic sequence *AB-delay-ab* is shown in the top panel of Fig. 2A. The input spike times are not kept fixed but newly drawn for each pattern presentation. This input encoding adds extra variability to the task, which is not directly reflected by the simple symbolic finite state grammar. Still, the statistics underlying the input sequences 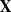 follow the dynamics of a HMM of the form (4), and therefore our WTA circuit and the spike trains that encode sequences generated by the artificial grammar share a common underlying model.

The observation model 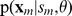 of that HMM covers the uncertainty induced by the noisy rate patterns by assigning a certain likelihood to each observed input activation 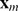. The hidden state representation has to encode the context-dependent symbol identity and the temporal structure of the sequences, i.e. the duration of each individual symbol. In our continuous-time formulation the hidden state is updated at the time points 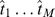. Therefore, throughout the presentation of a rate pattern of 50 ms length, several state updates are encountered during which the hidden state has to be maintained. In principle this can be done by allowing each hidden state to persist over multiple update steps by assigning non-zero probabilities to 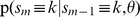. However, this approach is well known to result in a poor representation of time as it induces an exponential distribution over the state durations, which is inappropriate in most physical systems and obviously also for the case of deterministic pattern lengths, considered here [17], [19]. The accuracy of the model can be increased at the cost of a larger state space by introducing intermediate states, e.g. by representing pattern *B* in sequence *AB-delay-ab* by an assembly of states 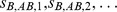 that form an ordered state sequence throughout the pattern presentation. Each of these assemblies encodes a specific input pattern, the temporal context and its sequential structure throughout the pattern, and with sufficiently large assemblies the temporal resolution of the model achieves reasonable accuracy. We found that this coding strategy emerges unsupervised in our WTA circuits through the STDP rule (7).

To show this, we trained a WTA circuit with 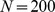 afferent cells and 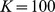 circuit neurons by randomly presenting input spike sequences until convergence. In this experiment, the patterns were presented as a continuous stream of input spikes, without intermediate pauses or resetting the network activity at the beginning of the sequences. Training started from random initial weights, and therefore the observation and prediction model had to be learned from the presented spike sequences. Prior to learning the neural activity was unspecific to the patterns and their temporal context (see Fig. 2B). Fig. 2C shows the evoked activities for all four sequences after training. The output of the network is represented by the perievent time histogram (PETH) averaged over 100 trial runs and a single spike train that is plotted on top. To simplify the interpretation of the network output we sorted the neurons according to their preferred firing times (see Methods). Each sequence is encoded by a different assembly of neurons. This reflects the structure of the hidden state that underlies the task. Since the input is presented as continuous spike train, the network has also learned intermediate states that represent a gradual blending between patterns. About 25 neurons were used to encode the information required to represent the hidden state of each sequence.

This coding scheme installs different representations of the patterns depending on the temporal context they appeared in, e.g. the pattern *delay* within the sequence *AB-delay-ab* was represented by another assembly of neurons than the one in the sequence *BA-delay-ba*. Small assemblies of about five neurons became tuned for each pattern and temporal context. This sparse representation emerged through learning and is not merely a consequence of the inherent sparseness of the WTA dynamics. Prior to learning all WTA neurons are broadly tuned and show firing patterns that are unordered and nonspecific (see Fig. 2B). After learning their afferent synapses are tuned for specific input patterns, whereas the temporal contexts in which they appear are encoded in the excitatory lateral synapses. The latter can be seen by inspecting the synaptic weights 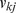 shown in Fig. 2D. They reflect the sparse code and also the sequential order in which the neurons are activated. They also learned to encode the stochastic transitions at the beginning of the cue phase, where randomly one of the four sequences is selected. These stochastic switches are reflected in increased strength of synapses that connect neurons activated at the end and the beginning of the sequences.

**Figure 3:**
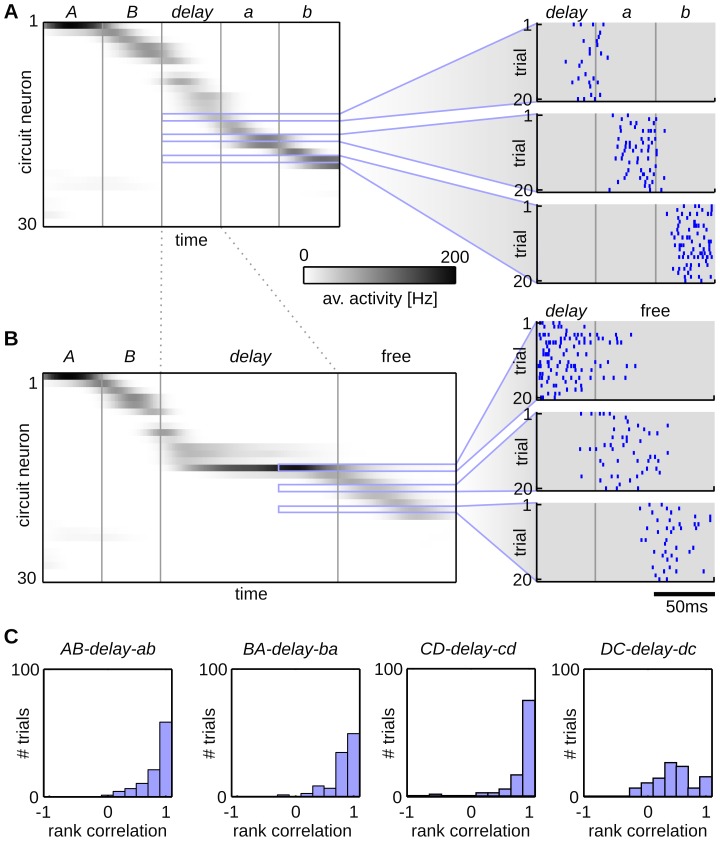
Spontaneous replay of pattern sequences. (A,B) The output behavior of a trained network for sequence *AB-delay-ab*. The network input is indicated by pattern symbols on top of the plot and pattern borders (gray vertical lines). (A) The average firing behavior of the network during evoked activity. The 30 circuit neurons that showed highest activity for this sequence are shown. The remaining neurons were almost perfectly silent. The network activity is averaged over 100 trial runs and neurons are sorted by the time of maximum average activity. Detailed spiking activities for three example neurons that became active after the delay pattern are shown. Each plot shows 20 example spike trains. (B) Spontaneous completion of sequence *AB-delay-free*. After presenting the cue sequence *AB* and the delay pattern for 150 ms the afferent input was turned off, letting the network run driven solely by lateral connections. During this spontaneous activity, the neurons are activated in the same sequential order as in the evoked trials. Detailed spiking activity is shown for the same three example neurons as in (A). (C) Histograms of the rank order correlation between the evoked and spontaneous network activity for all four sequences, computed over 100 trial runs. The sequential order of neural firing is reliably reproduced during the spontaneous activity and thus the structure of the hidden state is correctly completed.

The behavior of the circuit is further examined in Fig. 3. The average network activity over 100 trial runs of the neurons that became most active during sequence *AB-delay-ab* are shown in Fig. 3A. In addition the spike trains for 20 trials are shown for three example neurons. The same sorting was applied as in Fig. 2. Using the hidden state encoded by the network it should be possible to predict the recall patterns after seeing the cue, if it correctly learned the input statistics. We demonstrate this by presenting incomplete inputs to the network. After presentation of the delay pattern the input was turned off and the network was allowed to run freely. The delay pattern was played three times longer than in the training phase 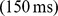. During this time the network was required to store its current state (the identity of the cue sequence). After this delay time the input was turned off – no spikes were generated by the afferent neurons during this phase, the network was purely driven by the lateral connections. Since the delay time was much longer than the EPSP windows the network had to keep track of the sequence identity in its activity pattern throughout this time to solve the task. Fig. 3B shows the output behavior of the network for sequence *AB-delay-free* (where *free* denotes a 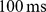 time window with no external input). After the initial sequence *AB* was presented, a small assembly of neurons became active that represents the delay pattern that was associated with that specific sequence. After the delay pattern was turned off, the network completed the hidden state sequence using its memorized activity, which can be seen by comparing the evoked and spontaneous spike trains in Fig. 3A and B, respectively.

In order to quantify the ability of the network to reproduce the structure of the hidden state, we evaluated the similarity between the spontaneous and evoked network activity using the rank order correlation coefficient, which is a similarity measure normalized between 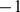 and 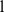, where 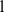 means that the order is perfectly preserved. This measure has been previously proposed to detect stereotypical temporal order in neural firing patterns [6]. Fig. 3C shows the histograms over the correlation coefficients for all four sequences. The histograms were created by calculating the rank order correlation between the spontaneous sequences and the PETH of the evoked sequences. It can be seen that the temporal order of the evoked sequence was reliably reproduced during the free run. To that end, for each of the input sequences, a stable representation has been trained into the network, that is encoded in the lateral synapses. This structure emerged completely unsupervised using the local STDP rule, solely from the intrinsic dynamics of the network.

### Mixed selectivity emerges in multiple interconnected WTA circuits

The first experiment demonstrated that through STDP, single neurons of a WTA circuit get tuned for distinct input patterns and the temporal context in which they appear. The neural code that emerged is reminiscent of some features found in cortical activity of monkeys solving similar tasks, namely the emergence of context cells that respond specifically to certain symbols when they appear in a specific temporal context [34], [36], [37]. However, the overall competition of a single WTA circuit hinders the building of codes for more abstract features, which are also found in the cortex in the very same experiments where neurons in the same cortical area encode different functional aspects of stimuli and actions. They seem to integrate information on different levels of abstraction which results in a diverse and rich neural code, where close-by neurons are often tuned to different task-related features [25].

We show that our model reproduces this mixed selectivity of cortical neurons if multiple interconnected WTAs are trained on a common input. The strong competition is restricted to neurons within every single WTA, whereas there is no competition between neurons of different circuits and lateral connections allow full information exchange between the circuits. Therefore, the model is extended by splitting the network into smaller WTA groups, each of which receives input from a distinct inhibitory feedback loop that implements competition between members of that group. In addition all neurons receive lateral excitatory input from the whole network. Every WTA group still follows the dynamics of a forward sampler for a HMM. Each of these WTA circuits adapts its synaptic weights through STDP to best represent the observed input spike sequences. In addition, the lateral connections between WTA groups introduce a coupling between the network states of individual groups. The dynamics of the whole network of WTA circuits can be understood as a forward sampler for a coupled HMM [38], where every WTA group encodes one multinomial variable of a compound state such that from one time step to the next all single state variables have influence on each other [20], [38].

**Figure 4:**
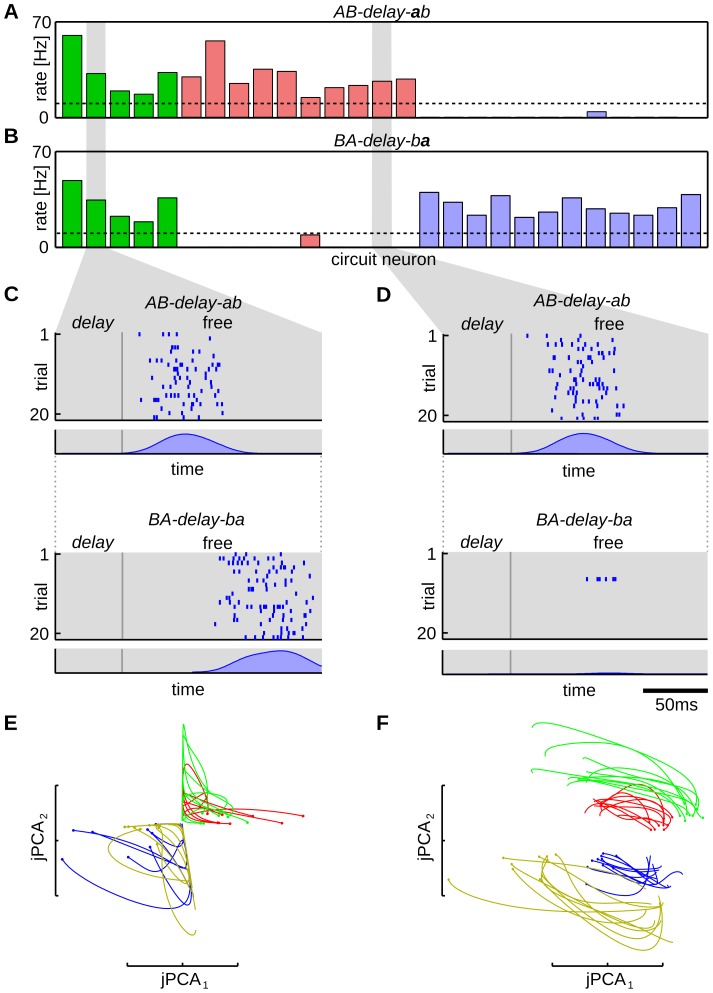
Mixed selectivity in networks of multiple interconnected WTA circuits. (A,B) Mean firing rate of the circuit neurons for evoked activity during pattern *a* in sequence *AB-delay-ab* (A) and *BA-delay-ba* (B). A threshold of 10 Hz (dashed line) was used to distinguish between neurons that were active or inactive during the pattern. Firing rates of neurons that were not context selective are shown in green, that of neurons selective for starting sequences *AB* and *BA* are shown in red and blue, respectively. Neurons that did not fall in one of these groups are not shown. Spike trains of one context selective (C) and one non-selective (D) neuron are presented for spontaneous completion of sequence *AB-delay-ab* (upper) and *BA-delay-ba* (lower) (cue phase is not shown). Spike raster plots over 20 trial runs and corresponding averaged neural activity (PETH) are shown. The two neurons encode the input on different levels of abstraction. The neuron in panel (D) shows context cell behavior, since it encodes pattern *a* only if it occurs in the context of sequence *ab*. During *ba* it remains (almost) perfectly silent. The neuron in (C) is not context selective, but nevertheless fires reliably during the time slot of pattern *a* during the free run by integrating information from other (context selective) neurons. It belongs to a WTA circuit with 15 neurons, for which the network state projection is shown in panel (E). (E,F) Linear projection of the network activity during the delay phase to the first two components of the jPCA, for a single WTA circuit with 15 neurons (E) and for the whole network (F). 10 trajectories are plotted for each sequence (*AB-delay-ab* red, *BA-delay-ba* green, *CD-delay-cd* blue, *DC-delay-dc* yellow). The dots at the beginning of each line, indicate the onsets of the delay state, i.e. the beginning of the trajectories. The plots have arbitrary scale. The projection of the WTA circuit in (E) does not allow a linear separation between all four sequences, whereas the activity of the whole network (F) clusters into four sequence-specific regions. The network neurons use this state representation to modulate their behavior during spontaneous activity.

In the first experiment we have seen that the WTA circuit learned to use about 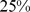 of the available neurons to encode each of the four sequences. We have also seen that the network used small assemblies of neurons to represent each of the patterns in favor of a finer temporal resolution. This implies that WTA circuits of different size can learn to decode the input sequence on different levels of detail, where small circuits only learn the most salient features of the input sequences. To show this we trained a network with 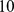 WTA groups of random size between 10 and 50 units, giving a total network size of 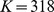, on the simple object sequence memory task (Fig. 2A). The neural code that emerges in this network after training is shown in Fig. 4. The output rates of the circuit neurons were measured during the presentation of pattern *a* appearing in the sequence *AB-delay-ab*, *BA-delay-ba*, shown in Fig. 4A,B respectively. Three classes of neurons can be distinguished: 10 neurons were tuned to pattern *a* in the context *AB-delay-ab* only (shown in red), 12 neurons were tuned to pattern *a* exclusievly in the context *BA-delay-ba* (shown in blue) and 5 additional neurons encode pattern *a* independent of its context (green), i.e. they get activated by the pattern *a* in both sequences *AB-delay-ab* and *BA-delay-ba*. The remaining neurons were not significantly tuned for pattern *a* (average firing rate during pattern *a* was less than 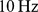, not shown in the plot).

To pinpoint the computational function that emerged in the network we compared the spontaneous activity of individual neurons from different WTA circuits. Spike trains for one context-specific and one non-specific neuron are compared in Fig. 4C and D, respectively. Both panels show spike raster plots over 20 trial runs and averaged neuron activities (PETH) for sequences *AB-delay-free* and *BA-delay-free*. The neuron in Fig. 4C belongs to a small WTA group with a total size of 15 neurons and shows context unspecific behavior, whereas the neuron in Fig. 4D which belongs to a larger WTA group (42 neurons) is context specific (see Fig. 4A,B). This behavior is also reproduced during the free run, when the neurons are only driven by their lateral synapses. The neuron in Fig. 4D remains silent during *BA-delay-free* and thus shows the properties of context cells observed in the cortex, whereas the neuron in Fig. 4C is active during both sequences. Still, during spontaneous replay that neuron correctly reproduces the temporal structure of the input sequences. In sequences starting with *AB* the neural activity peaks at 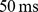 after the onset of the free run – the time pattern *a* was presented in the evoked phase. If the sequence starts with *BA* this behavior is modulated and the activity is delayed by roughly 50 ms, to the time point *a* would appear in the recall phase. The required information to control this modulation was not available within the small WTA group the neuron belongs to, but provided by neighboring context-specific neurons from other groups.

To see this we trained a linear classifier on the evoked activity during the delay phase of *AB-delay-ab* and *BA-delay-ba* (see Methods for details). If the neurons reliably encode the sequence identity a separating plane should divide the 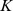-dimensional space of network activities between the sequences. Training the classifier only on the 15-dimensional state space of the group the neuron in Fig. 4C belongs to, did not reveal such a plane (the classification performance was 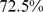). Therefore, this small WTA circuit did not encode the required memory item to distinguish between the two sequences after the delay phase. However, the whole network of all WTA groups reliably encoded this information and the classifier trained on the 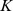-dimensional state space could distinguish between the delay phases of *AB-delay-ab* and *BA-delay-ba* with 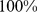 accuracy.

To illustrate the different emergent representations, we compared linear projections of the state of the small WTA group with 15 neurons and the state of the whole network in Fig. 4E,F, respectively. The plots show the network activity during the delay phase for all four sequences. Each line corresponds to a trajectory of the evoked network activity, where the line colors indicate the sequence identity. The state trajectories were projected onto the first two dimensions of the dynamic principal component analysis (jPCA), that was recently introduced as an alternative to normal PCA that is applicable to data with rotational dynamics [39]. Empirically, we found this analysis method superior to normal PCA in finding linear projections that separate the network states for different input sequences. One explanation for this lies in the dynamical properties of WTA circuits. Due to the global normalization which induces a constant network rate, the dynamics of the network are roughly energy-preserving. Since this implies that the corresponding linear dynamical system is largely non-expanding/contracting, a method that identifies purely rotational dynamics such as the jPCA was found to be beneficial here.

Fig. 4E shows the first two jPCA components of the neural activities during the delay phase for the WTA circuit with 15 neurons, which the neuron in Fig. 4C belongs to. This circuit was not able to distinguish between all four input sequences, since it activated the same neurons to encode them. This is also reflected in the jPCA projections shown in Fig. 4E, which show a large overlap for sequences *AB-delay-ab* and *BA-delay-ba*. On the other hand, the network state comprising all 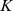 neurons reliably encoded the sequence identities (see Fig. 4F). The delay state for each sequence spans an area in the 2-D projection and therefore the network found a state space that allows a linear separation between the sequences. Such a representation is important since the neuron model employs a linear combination of the network state in the membrane dynamics (1) and therefore provides the information required by the neurons in Fig. 4C,D to modulate their spontaneous behavior.

### Trajectories in network assemblies emerge for stationary input patterns

Information about transient stimuli is often kept available over long time spans in trajectories of neural activity in the mammalian cortex [5], [7], [26], [40] and in songbirds [41]–[43]. In the previous experiment we saw that our model is in principle capable to develop such trajectories in neural assemblies (see Fig. 3B), which emerged to encode salient input patterns and the temporal structure throughout them. However, in that experiment the input sequences comprised a rich temporal structure, since each pattern was only shown for a 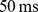 time bin which might have facilitated the development of these activity patterns. In this section we study whether a similar behavior also emerges when the input signal is stationary over long time spans.

**Figure 5:**
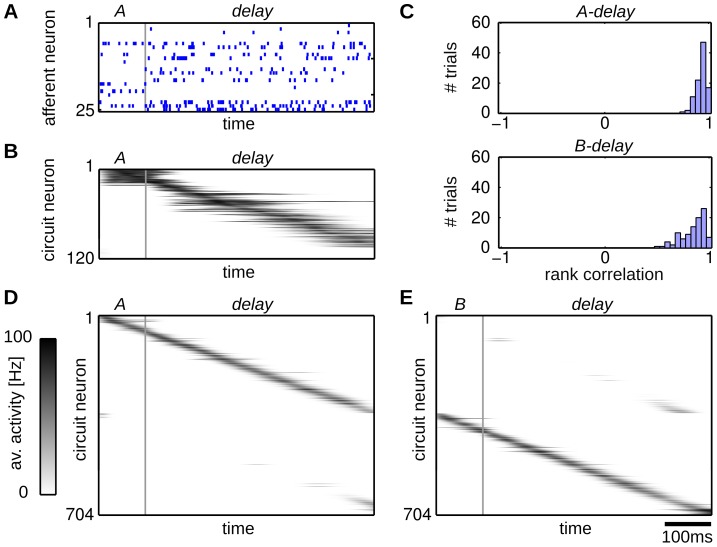
Neural trajectories emerge for stationary input patterns. (A) A network was trained with an extended delay phase of 500 ms. Input spike trains of a single run for sequence *A-delay* (25 out of 100 afferent neurons). Throughout the delay phase the afferent neurons fire with fixed stationary Poisson rates. (B) The output behavior for sequence *A-delay* averaged over 100 trial runs. The circuit neurons are sorted according to their mean firing time within the sequences (120 out of 704 neurons are shown). (C) Histograms of the rank order correlation between the evoked and spontaneous network activity. The sequential order of neural firing is preserved during spontaneous activity. (D,E) Homeostatic plasticity enhances the formation of this sequential structure. The output behavior of the network trained with STDP and the homeostatic plasticity mechanism is shown. Approximately 50% of the neurons encode each of the two sequence. The neurons learn to fire at a specific point in time within the delay patterns, building up stable trajectories.

In analogy to the previous experiment we generated two input sequences *A-delay* and *B-delay*. The patterns *A*, *B* were played for 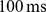 and the pattern *delay* for 500 ms. As in all other experiments, the patterns were rate patterns, i.e. each input neuron fired with a constant Poisson rate during the pattern and spike times were not kept fixed throughout trials. One example input spike train is shown in Fig. 5A.

Although the input was stationary for 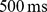 during the *delay* pattern, we could still observe the emergence of neural trajectories in the network after training. Again, we used a network composed of multiple interconnected WTA circuits to learn these patterns. We employed a network of 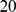 WTA groups of random size in the range from 10 to 100 neurons. The total network had a size of 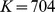 circuit neurons and we used 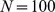 afferent cells. Fig. 5B shows the sorted average output activity after training. For each of the two sequences a distinct assembly of neurons emerged and the neurons composing these assemblies fired in a distinct sequential order. Fig. 5C shows the rank order correlations between the evoked and spontaneous activities. The trajectories of neural firing were reliably reproduced during spontaneous activity, but only about 100 neurons were used for each of the two assemblies, leaving the remaining 500 neurons (almost) perfectly silent.

The emergence of these trajectories can be further enhanced using a homeostatic intrinsic plasticity mechanism which enforces that on average all network neurons participate equally in the representation of the hidden state. This can be achieved by a mechanism that regulates the excitability 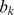 of each neuron, such that the overall output rate 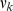 of neuron 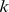 (measured over a long time window) converges to a given target rate 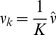. (see [44] and the Methods section). Augmenting the dynamics of the network with this intrinsic plasticity rule prevents neurons from becoming inactive if their synaptic weights decrease and by that assures that each neuron joins one of the assemblies. This can be seen in Fig. 5C,D which shows the output activity after training with STDP augmented with the homeostatic mechanism. The neurons formed a fixed ordered sequence and thus showed a clear preference for a certain point in time within the pattern. Even though the delay pattern had no salient temporal structure (the rates of all afferent neurons were constant throughout the pattern) these trajectories were formed by imprinting the sequential order of the neural activity into the lateral excitatory connections. As in the first experiment each neuron has learned to fire after a distinct group of preferred predecessors, resulting in neural trajectories through the network. Therefore, the time that has elapsed since the *delay* pattern started could be inferred from the neural population activity. In addition the identity of the initial pattern was also memorized, since about half of the population became active for each of the two sequences.

### Learning the temporal structure of an artificial grammar model

**Figure 6:**
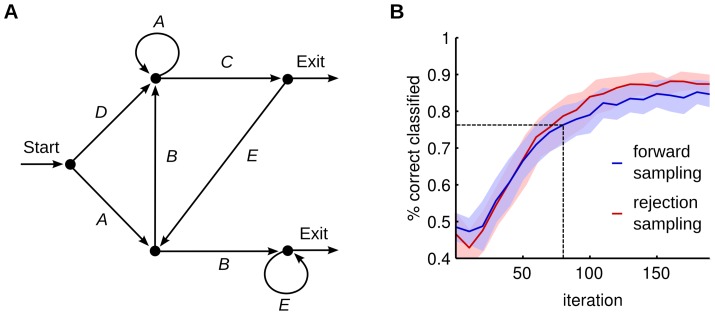
Fast learning of an artificial grammar. (A) The artificial grammar from [27], [74] represented as a finite state grammar graph. Grammatical sequences are generated by following a path from *Start* to *Exit*. If a node has more than one outgoing arc one is chosen at random with equal probability to continue the path. (B) Convergence of the network performance on that task. The blue curve shows the evolution of the mean classification performance against the number of training samples, when forward sampling was used. The blue shaded area indicates the standard deviation over 20 trial runs. After 80 training samples the network exceeds human performance reported in [27]. Using rejection sampling with 10 samples on average (red curve) does not significantly outperform forward sampling on this task.

The finite state grammar used in the previous experiments (Fig. 2A) did not utilize the full expressive power of HMMs since it only allowed stochastic switches at the beginning of each sequence. In this section we consider the problem of learning more general finite state grammars in WTA circuits, a problem that has also been extensively studied in cognitive science in artificial grammar learning (AGL) experiments [45]. Fig. 6A shows the artificial grammar that was used in [27] to train subjects using different stimulus modalities (visual, auditory and tactile). There it was shown that humans can acquire the basic statistics of such grammars extremely fast. On this particular task humans showed a performance of 62% to 75% percent (depending on the stimulus modality that was used) after only a few dozens of stimulus presentations [27].

We show that our network model can extract the basic structure of this grammar. This internal representation can be subsequently used to classify unseen sequences as grammatical or not. Through STDP the network adapts the parameters 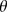 such that they reflect the statistics underlying the training sequences, and the emergent HMM can then be used to evaluate the sequence likelihood 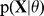. The ability of the network to distinguish between grammatical and ungrammatical sequences was assessed by applying a threshold on the sequence log-likelihood, an approximation of which was computed over a single sample 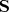 from (5) (see Methods). The threshold was assigned to the mean of the log-likelihood values computed for all test sequences. Likelihoods that laid above that threshold were reported as grammatical.

In this experiment we used a sparse input coding, where only a small subset of afferent neurons is activated for each of the symbols. This representation could be realized by another WTA circuit used as input for the network to decode more complex input patterns. We trained a single WTA circuit with 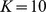 neurons on this sparse input. Using this model, we were able to achieve high learning speeds. In each training iteration one of the 12 training data sets from [27] (using only the first sequence of each match/mismatch pair) was chosen at random and presented to the network. For testing we used the 20 test sequences from [27] to evaluate the learning performance. Training was interrupted after every 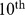 sequence presentation to assess the classification performance. The resulting learning curve is shown in Fig. 6B. The classification rate of 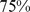 that was reported in the behavioral experiment was exceeded after only 80 iterations. By training the network beyond this point performances up to 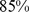 were reached. Note that none of the training sequences appeared in the test set. Therefore the network has not just learned a fixed set of sequences, but extracted relevant statistical features that allowed it to generalize to new data.

### A refined EM approximation using rejection sampling

So far in all experiments the simple forward sampling approximation was used for learning the model parameters. Although this learning paradigm has shown to be surprisingly powerful, it is limited and will not be sufficient if the network is required to learn more complex tasks or acquire probabilistic models with a high level of detail. In this section we derive the refined approximation toward evaluating the HMM E-Step in a recurrent WTA circuit based on rejection sampling.

Exactly solving the E-step requires to evaluate the posterior probability of 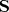, given by
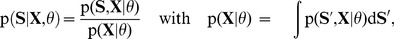
(8)where 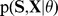 is the HMM joint distribution, given by equation (4). A stochastic EM update is realized by drawing a state sequence from the posterior for which the M-step parameter updates are performed. However, directly sampling from (8) is not possible for a spiking neural network, since it requires the integration of information over the whole state sequence and thus, looking into the future. This can be seen by noting that the integral in (8) runs over the state space of the whole sequence. To that end, the network is not able to sample from this distribution directly. Nevertheless, it is possible to indirectly evaluate (8) using samples generated from (5), which can be expressed by
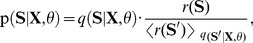
(9)where 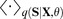 denotes the expected value over 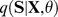, which in this context is called a *proposal distribution* since it is used to propose samples, which are then used to indirectly evaluate the target distribution 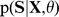. The scalar 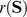 is the *importance weight* between the target and the proposal distribution, which is used to scale the influence of the sample 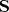 [19], [20], [46].

The expectation in the denominator of (9) is again not easy to evaluate, since it requires us to integrate over multiple sequences. The most pragmatic solution to this problem is to approximate this term using a single sample from the proposal distribution 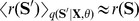. Under this approximation the importance weight in (9) cancels out and we arrive at the trivial approximation 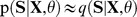, i.e. each sample from the proposal distribution is accepted as a valid sample from the posterior. This is the forward sampling approximation that was used so far throughout all experiments.

In order to improve this approximation we use the stochasticity of the network, which assures that different state sequences 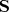 are proposed if the same input sequence 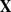 is presented several times. Rejection sampling utilizes this stochasticity and preferentially selects sequences with high likelihood throughout the whole input. The required information to do this selection is a global quantity that must be tracked over the whole sequence. The probability to accept a state sequence 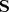 is directly proportional to the importance weight 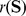, which computes to
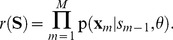
(10)Note that (10) can be easily computed forward in time, since in each time step, it only needs to be updated using the instantaneous input likelihood 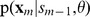. Further note that this is a measure for surprise or prediction error – the probability of observing the current input given the previous state. The information to decide whether to accept 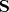 is the accumulated prediction error over the whole sequence. This approach also naturally extends to the case of multiple interconnected WTAs. There, the contributions to the importance weight of every single circuit have to be multiplied in every time step and therefore, a possible rejection is in that case effective for the whole network of all WTAs at once.

Since the importance weights need to be accumulated over the whole sequence of spike events of length 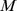, the weight update rules (7) can not be applied instantaneously. In the neural implementation we achieved this using a synaptic eligibility trace as proposed in [47]. Instead of updating the weights directly they are tagged and consolidation of the tags is delayed until the whole sequence is read. The probability to accept these tags is proportional to the importance weights, i.e.
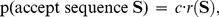
(11)where 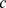 is a constant that scales the acceptance rate. If a sequence 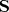 is accepted, the synaptic tags are consolidated. If the circuit decides not to accept, the synaptic weight changes for the whole sequence have to be discarded. This result is analytically similar to [15], where the importance weights (10) were introduced by weighting the eligibility traces with a deterministic scalar factor (importance sampling). Here, in the rejection sampling framework a stochastic variant of this method is used. The advantage of the rejection sampling method is that it is not necessary to explicitly compute the normalization in (8). The normalization can be approximated by replaying in every training iteration the input sequence multiple times until it gets accepted once, instead of using a constant number of replays as with importance sampling. In practice however it is necessary to adapt the parameter 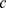 throughout learning in order to get a reasonable number of replays. We used a simple linear tracking mechanism for 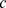 throughout the experiments (see Methods). A performance comparison of these different sampling approximations is provided at the end of the Results section.

We assume that the circuit interacts with a mechanism that allows the replay of the afferent stimulus multiple times. By enforcing that each input is accepted once, we guarantee that the network learns the statistics of all input sequences with equal accuracy. This view allows us to make an interesting theoretical prediction: when an input is not well represented by the network it is more likely to be rejected and therefore, the number of rejected and resampled sequences represents a notion of novelty. Literally speaking, the network pays more attention to novel inputs, by resampling them multiple times (see Methods for details).

### Rejection sampling enhances the learning capabilities of STDP

In the following experiments we investigate the possible performance gain that can be achieved if the network has access to this rejection sampling mechanism. We have previously seen that the grammar from Fig. 1 in [27] can be learned almost perfectly using pure forward sampling. However, this data set had a very simple structure. To distinguish between grammatical and ungrammatical sequences only required the analysis of the local statistics of the input. E.g. it is easy to see that the sequence *DEAC* is not grammatical since it contains the bigram *DE*, which never appears in the training data. Each of the ungrammatical sequences contains at least one illegal bigram and thus can be classified based on a simple model of symbol transitions. This simple structure was already recovered with the online learning scheme and therefore using rejection sampling on that task did not result in a significant performance increase (see Fig. 6).

**Figure 7:**
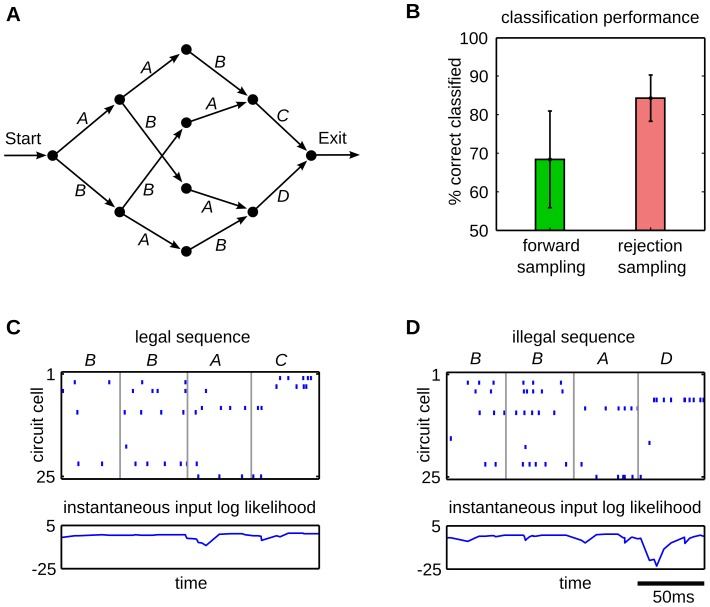
Rejection sampling enhances the classification performance of the network. (A) The grammar graph used for this task. A three letter sequence composed of *A*s and *B*s identifies the last symbol, *C* or *D*. Therefore, the most salient information is provided at the end of the sequence. (B) The classification rate on this task is plotted for forward (green) and rejection sampling (red). The error bars indicate the standard deviation over 10 trial runs. Rejection sampling significantly increases the classification performance on this task. (C,D) Comparison of the time courses of the instantaneous input log likelihood for a legal input sequence *BBAC* (C) and an illegal sequence *BBAD* (D). Input patterns are indicated by the pattern symbols on top of the plots. The upper plot shows the output spike trains of the network, the lower plot shows the traces of the instantaneous input likelihood plotted in the log domain, which indicates the ability of the network to predict the continuation of the afferent spike train. The trace in (D) shows a strong negative peak at the illegal transition at 150 ms. The prediction model that emerged through STDP augmented with rejection sampling, enables the network to detect illegal sequences.

To demonstrate the advantage of rejection sampling, we created a grammar that required integration of information over a longer time span, shown in Fig. 7A. Although this grammar only allows to create four sequences *AABC*, *BBAC*, *ABAD* and *BABD*, the underlying structure is more complex than in the previous tasks. The identity of the last symbol can only be inferred if the identity and context of the first symbol is integrated and memorized over the whole sequence. To that end, the rejection sampling algorithm that allows the network to propagate information over the whole sequence, should bring a definite benefit over forward sampling for this task.

The quantity that is needed to update the importance weights (10) and also to estimate the sequence likelihood for classifying grammatical against ungrammatical inputs, is given by the instantaneous input likelihood 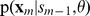 (see Methods). As pointed out earlier, this quantity is a measure for surprise, i.e. the probability of observing the current input pattern given the network state. The ability of the network to exploit this prediction error to classify sequences is illustrated in Fig. 7. The input-output behavior of a network after training with rejection sampling is shown for the grammatical sequence *BBAC* and the ungrammatical sequence *BBAD*, in Fig. 7C,D respectively. The bottom plots show traces of the instantaneous input log-likelihood. Throughout the grammatical sequence in Fig. 7C the trace stays near baseline, which indicates that the network is capable of predicting the sequence. Within the patterns, the trace only shows small deviations due to input noise. Switches between the input patterns e.g. at the border from pattern *A* to *C* cause modest levels of surprise, due to the sudden change of the network state. However, the illegal transition to pattern *D* in Fig. 7D causes a strong negative peak. At this point the network is not capable of predicting the final pattern. Thus the input is assigned to a low overall sequence likelihood and will therefore be classified as ungrammatical.

In the rejection sampling algorithm this quantity is also used throughout training, to learn preferably from sequences that are best capable of predicting the input sequences. To quantify the advantage of this method over online learning we compared the performance on the AGL task. As in the previous experiment, the ability of the network to distinguish between grammatical and ungrammatical sequences was evaluated by applying a threshold on the sequence likelihood. The threshold was assigned to the mean of the log-likelihood values computed for all tested sequences. The network parameters were tuned such that the number of rejected samples in each iteration, averaged over the whole training session was equal to the desired number of samples (see Methods). The classification errors are compared in Fig. 7B for learning with forward and rejection sampling. The parameter 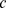 that scales the number of rejected samples was tracked to give an average number of 10 rejected samples per iteration. Despite this relatively small number of times the sequences is resampled, it can be seen that the performance on this task significantly increased with rejection sampling. Online learning achieved a classification rate of 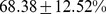. With rejection sampling the network achieved 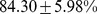 classification rate. Hence we confirmed, that having access to the rejection sampling mechanism allows the network to learn the input statistics with higher levels of accuracy. Furthermore, for the example given here, this was achieved with a relatively small average number of resampled state sequences.

### Comparison of the convergence speed and performance of the approximate algorithms

In order to give a quantitative notion of how the sampling approximations affect the learning performance, we applied the methods to solve a generic HMM learning task. To allow a direct comparison with standard machine learning algorithms for HMMs, we used a time-discrete version of our model in this section. Therefore, we set the inter-spike-intervals 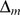 to a fixed constant value and used rectangular EPSP kernels of the same length. With this modification our model is equivalent to a discrete input, discrete state HMM, commonly considered in the machine learning literature [19]. We created random HMMs and used them to generate a training and a test data set. Using this data we compared the training performance of different approximation algorithms.

The accuracy of the rejection sampling algorithm crucially depends on how the parameter 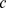 in equation (11) is selected. If it is set to a very large constant value, every sample gets accepted and we arrive at the simple forward sampling approximation. We compared this forward sampling algorithm with the simple tracking algorithm that was used in the previous experiment and with the optimal mechanism, which computes 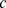 over a batch of sampled sequences (see Methods). In addition we compared these methods with the importance sampling algorithm considered in [15], where the scalar values of the importance weights were directly used to weight synaptic tags. All sampling methods were compared for an average number of 10 and 100 resampled sequences. Furthermore we applied standard EM learning for HMMs (the Baum-Welch algorithm) as reference method [19], [31].

**Figure 8:**
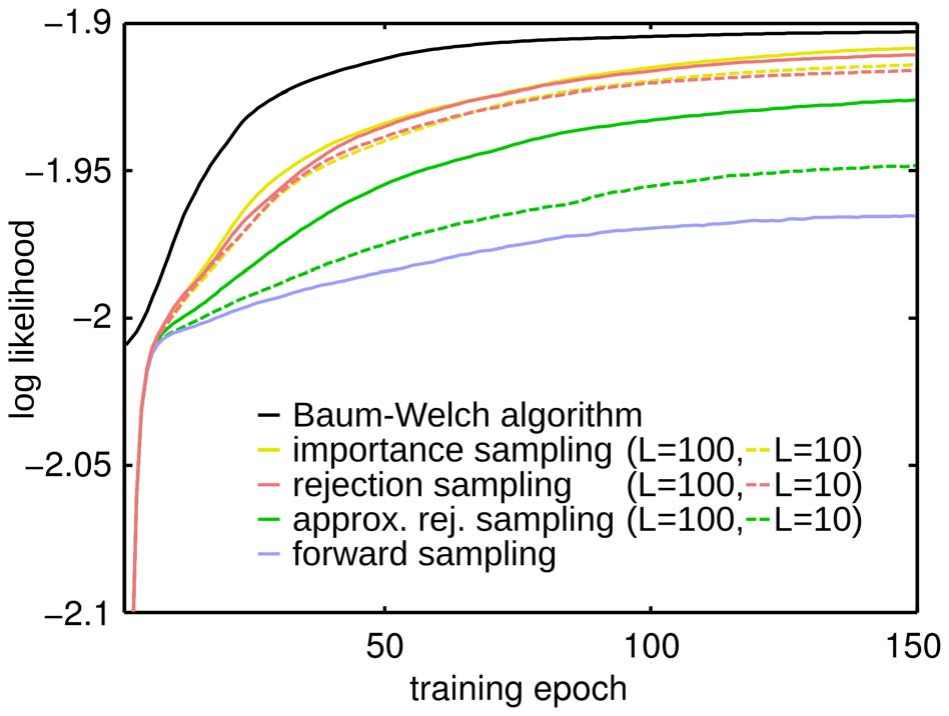
Comparison of the convergence speed and learning performance of different sampling methods. Comparison of the sampling approximations to standard HMM learning. The performance is assessed by the log likelihood averaged over 50 trial runs. The plots show average convergence properties of: forward sampling (solid blue), importance sampling over 10 (dashed yellow) and 100 trials on average (solid yellow), rejection sampling over 10 (dashed red) and 100 trials (solid red), rejection sampling with the simple linear tracking of 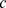 over 10 (dashed green) and 100 trials on average (solid green), and the Baum-Welch algorithm (solid black). With increased number of samples the performance of the algorithm converges towards the solution of the standard EM algorithm. There was no significant performance difference between rejection and importance sampling. The simple tracking mechanism for the rejection sampler is outperformed by the exact algorithm, but still a significant performance gain with increased number of samples can be observed.

The results of the eight different training algorithms are compared in Fig. 8. The figure shows the log-likelihood on the test data averaged over the 50 learning trials. As can be seen, pure forward sampling shows poor performance on this task compared with Baum-Welch learning, but with increasing number of samples the approximation approaches the performance of the exact EM updates. Interestingly we found that importance sampling and rejection sampling show almost the same performance. We believe that the reason for this lies in the high variance of the importance weights. The weights of consecutive samples can differ several orders of magnitude. After normalization, effectively only the sample with the highest importance weight has non-zero influence on the weight updates. Therefore the two algorithms are numerically almost identical for the task considered here. Using the tracking mechanism for 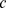 resulted in decreased performance compared to the exact algorithm. Still, a significant performance gain can be observed with increased average number of samples.

## Discussion

We have shown that STDP in WTA circuits with lateral excitatory connections implements the capability to represent the statistical structure underlying time-varying input patterns. The different types of excitatory synapses in the network serve different computational functions. Lateral connections recurrently feed back past network spikes which are used to predict a prior belief about the current network activity. The feedforward synapses match this prediction against the belief inferred from afferent inputs. The sparse code that emerges in this circuit allows to represent the activity of the whole network as samples from the state space of a HMM, implementing a forward sampler, which provides the circuit with a simple online approximation to exact HMM inference.

We have focused in this article on an idealized version of the STDP rule that implements the maximization step of the EM algorithm for the HMM. Similar rules also emerged in earlier studies as stochastic approximations to EM implemented in networks of spiking neurons [13], [32], [33], [48], for learning instantaneous afferent spike patterns. The only structural difference in the network architecture for temporal models is the presence of lateral excitatory connections. We have shown that if a WTA circuit is passively exposed to spatiotemporal rate patterns STDP implements a crude online approximation of EM. The emerging neural codes represent the hidden states that underlie the spatiotemporal input patterns. Different neurons are activated for the same input pattern if it appears in different temporal contexts. Furthermore, we have shown that if multiple WTA circuits are recurrently interconnected the network activity becomes more diverse and encodes various abstract features.

Throughout our analysis we realized the WTA dynamics using a feedback loop, where the required inhibition was given in its theoretically optimal form, according to equation (3). This optimal inhibition predicted by our model is strongly correlated with the activation of excitatory neurons within the WTA circuit. Such strong balance and correlation between excitation and inhibition has been observed in the cortex in vivo [2], [49]. A consequence of this inhibitory feedback in our network model is that the total output rate is constant. Yet individual neurons in the network may exhibit complex behavior, and our experimental results have shown that they exploit a wide dynamical range. Furthermore it has been shown in [13] that the assumption of a constant overall output rates can be lifted for the case of WTA circuits without lateral synapses. The only requirement identified there was that the circuit-wide output rate and the input had to be stochastically independent, which was theoretically shown and experimentally verified in an experiment where the network rate was modulated with a global oscillation throughout learning. The constant output rate considered in the present study is the simplest case which is compatible with our model. Identifying a more general class of rate functions which can be incorporated into our theoretical framework will be the subject of future work.

The activity patterns that emerge in our WTA circuits share important features with experimentally observed cortical dynamics. One feature is that neurons become tuned for mixtures of different task-relevant aspects as commonly observed in cortical neurons [25], [34], [36]. The neural assemblies that encode these temporal features imprint their stereotypical sequential activation pattern within the lateral synapses. Another common feature is the emergence of stereotypical firing sequences during evoked and spontaneous activity, which is also found in cortical activity [5], [7], [26]. This analysis also provides a theoretical foundation for the results that were reported recently in [50]. There, a similar network of stochastic WTA circuits was used to learn spike patterns superimposed with Poisson noise, and similar stimulus-specific assemblies emerged. But no theoretical framework was provided there.

The network of multiple interconnected WTA circuits has a very interesting theoretical interpretation as it implements in that case a forward sampler for a coupled HMM [38], where multiple HMMs run in parallel to jointly encode the hidden state. In our experiment this coupling between neighboring WTA circuits allowed them to reproduce typical sequences of hidden states in the absence of input, even if some circuits did not have enough expressive power to store this information. An interesting future extension of this model would be to present different coupled stimuli (e.g. speech and audio from a common source) to different WTA groups in this circuit. Individual WTA circuits would then learn the temporal structure of these stimuli and the lateral excitatory synapses between WTA circuits would detect relevant correlations between them.

We have also shown that STDP installs in WTA circuits capabilities that go beyond just learning afferent sequences. From few presentations the network extracted relevant statistical properties underlying the afferent patterns. We demonstrated this on an artificial grammar learning task. The network extracted parts of the structure of this grammar, which allowed it to subsequently classify unseen sequences as stemming from the same grammar or not. Interestingly, the learning speed and classification performance achieved with the forward sampling approximation, in the early learning phase, is comparable to the performance reported for humans on the same task [27]. This is also interesting because the network considered here is similar to the single recurrent network (SRN) previously suggested as a model for artificial grammar learning [51], [52]. The context layer, that is used in the SRN to store the hidden layer activity from previous time steps, is implicitly implemented in the lateral synapses of our WTA circuit. The SRN was successfully used to model human capabilities in artificial grammar learning tasks [53] (but see [54], [55], for alternative theories and models of artificial grammar learning).

We have also exhibited a strategy to increase the computational power of WTA circuits by using more advanced learning methods. The rejection sampling algorithm that was proposed here is one possible solution to this problem. It enables the network to learn the temporal statistics with a much higher degree of accuracy, but at the same time it considerably increases the complexity of learning. Each input sequence must be replayed multiple times and thus, the convergence speed is decreased since many sampled paths will be rejected (in the experiment we resampled each path 10 times on average, therefore the learning time increased 10-fold). This makes learning possible on a long time scale only. However, the two mechanisms – pure forward sampling and rejection sampling – should not be seen as mutual exclusive strategies. Possibly both mechanisms could be found in biological systems. STDP might subserve to learn a quick preliminary representation of novel input statistics, while more complex models could emerge on a long time scale by selectively modulating the learning rate with global information. We demonstrated that in some cases a significant increase in learning performance can be achieved with only a small average number of resampled sequences. The experimental results suggest that for learning temporal sequences and simple grammars the pure implementation of STDP in WTA circuits is sufficient, whereas third-factor STDP rules become relevant for learning complex temporal structures.

### Related work

The close relation between HMMs and recurrent neural networks was previously discovered and employed for deriving models for Bayesian computation in the cortex. These studies targeted the implementation of Bayesian filtering [56], [57], capturing the forward message of the belief propagation algorithm in a rate-based neural code, or using a two-state HMM to capture the dynamics of single neurons [58], [59]. In the present study we directly analyzed spikes produced by WTA circuits in terms of samples from the state space of a HMM. For the HMM this results in an arguably weaker form of inference than belief propagation, but led in a straightforward manner to an analysis of learning in the network.

The emergence of predictive population codes in recurrent networks through synaptic plasticity and their importance for sequence learning was previously suggested and experimentally verified [60], [61]. In [62] it was shown that spiking neurons can learn the parameters of a 2-state HMM using synaptic plasticity, thereby implementing an online EM algorithm [63], [64]. In [14] learning of temporal models was implemented through a variational approximation, and revealed STDP-like learning rules. In [15] it was shown that a network of neurons can learn to encode and reproduce a sequence of fixed spike times. The learning rules were derived using an importance sampling algorithm that yielded synaptic updates similar to the third-factor STDP rule presented here.

The crucial difference between [15] and our approach is the usage of WTA circuits as building blocks for the recurrent network instead of individual neurons. Due to the possibility to use multiple WTAs our model has the freedom to factorize the multinomial HMM state space into smaller coupled variables, whereas [15] always fully factorizes the state space down to single binary variables. However, under the assumption of linear neurons the state-transition probabilities in all these models are always represented by only 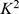 recurrent synapses. Thus the expressive power of all these models (with the same number of neurons) should be more or less identical. The optimal factorization of the state space may strongly depend on the task. Our experiments suggest that the restriction on the number of possible activity patterns due to the usage of WTAs seems minor compared to the crucial advantage of their intrinsic stabilizing effects of the network's activity. To the best of our knowledge this stabilization is the reason why the pure forward sampling learning approach performed so well in our experiments.

### Contribution to a principled understanding of computation and plasticity in cortical microcircuits

The theoretical framework that we have introduced in this article provides a new and more principled understanding for the role of STDP in a generic cortical microcircuit motif (ensembles of pyramidal cells with lateral excitation and inhibition): Even in the absence of global signals related to reward, STDP installs in these microcircuit motifs an approximation to a HMM through forward sampling. The underlying theoretical analysis provides a new understanding of the role of spikes in such WTA circuits as samples from a (potentially very large) set of hidden states that enable generic cortical microcircuits to detect generic neural codes for afferent spike patterns that can reflect their temporal context and support predictions of future stimuli.

A remarkable feature of our model is that it postulates that noise in neural responses plays a very important role for the emergence of such “intelligent” temporal processing: We have shown that it provides in WTA circuits the basis for enabling probabilistic inference and learning through sampling, i.e. through an “embodiment” of probability distributions through neural activity. Thus stochasticity of neural responses provides an interesting alternative to models for probabilistic inference in biological neural systems through belief propagation (see [65] for a review), i.e. through an emulation of an inherently deterministic calculation.

The rejection sampling algorithm that was proposed here as a method for emulating the full power of HMM learning requires in addition a mechanism that allows to replay input patterns multiple times. Such replay of complex spatiotemporal patterns is well documented in the hippocampus and was proposed as a mechanism for memory consolidation in the cortex [66]. This view is also supported by findings that showed that coordinated reactivation of temporal patterns can be observed in the cortex [8], [9], [67], [68]. In our framework, samples generated by the WTA circuit must be replayed several times until the network produces a spike train that provides a sequence of hidden states that gives satisfactory explanations and predictions for all segments of the sequence. The number of times a sequence is replayed is proportional to the prediction error accumulated over the sequence, which is a measure for the sample quality. Thus, sequences that are novel and to that end not well represented in the network should be replayed more often and thus, they get more attention in the learning process. This view is supported by experimental data that revealed that transient novel experiences are replayed more prominently than familiar stimuli [69]–[71].

Altogether our results show that hidden Markov models provide a promising theoretical framework for understanding the emergence of all-important capabilities of the brain to understand and predict hidden states of complex time-varying sensory stimuli.

## Methods

### Spiking network model

In this section we provide additional details to the derivations of the network model and its stochastic dynamics. For the sake of simplicity, throughout the theoretical analysis we use a simple EPSP kernel of the form
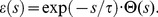
(12)Thus, a kernel with a single exponential decay with time constant 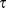. Here, 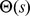 determines the Heaviside step function which is 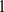 for 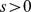 and zero else.

The derivation provided here can be extended to more complex EPSP shapes, if two prerequisites are fulfilled. First, a suitable Markov state must be found that describes the dynamics of the EPSP kernel, i.e. a state 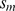 must exist for which we can write 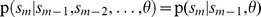. In fact, this property holds true for any deterministic function, although the required Markov state can be very complex. Second, the statistics of the EPSPs induced by the kernel must be readily described by an exponential family distribution. For this latter requirement the same considerations as for the afferent synapses apply, which have been addressed in [13], [32], [33]. The simplest case for which these conditions are fulfilled is the one considered in the last experiment where rectangular EPSPs and constant inter-spike intervals 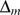 of the same length were used. In that case the network state collapses to 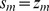, which follows a multinomial distribution as considered in [32].

#### Details to *Forward sampling in WTA circuits*

We show here that the WTA circuit correctly implements forward sampling in a HMM. In particular we show that a HMM with an observation model from the exponential family can be directly mapped to the network dynamics. Many of the theoretical details for the special case of stationary input patterns were analyzed in [13], [32], [33], here we focus on the derivations specific for the network with lateral excitatory connections.

First we define a HMM with observations 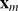 and hidden state 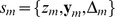 to reflect the dynamics of the WTA circuit. The HMM joint distribution is given by equation (4). Each time step 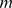 factorizes into the *observation model* 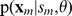 and the *prediction model* 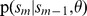. We assume a mixture of exponential family distributions for the observation model. Many interesting distributions are members of this family, e.g. the Poisson or the Normal distribution. The network output 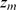 determines which mixture component is responsible for the observation 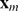. In its generic form the likelihood of the 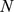-dimensional observations 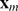 given mixture component 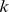 can be written as
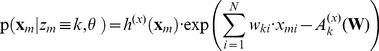
(13)where 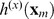 is a base measure and 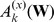 is the log-partition function, which assure that (13) is correctly normalized. In this framework 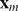 determines the sufficient statistics of the input distribution, e.g. the current input rate for the Poisson distribution, which is estimated by filtering the input spike train with the EPSP kernel. Since the input and output spike times are independent, given optimal WTA behavior, we exploit the conditional independence 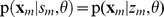. We assume that inputs are homogeneous, meaning that the sums over all input channels are constant. More precisely, we assume that 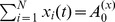 and 
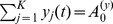
 holds true at all times. These assumptions were never perfectly fulfilled in the simulations, but nevertheless the algorithm was robust against deviations from these constraints throughout all experiments. The choice of the log-partition function determines the member from the exponential family. The derivations here were done for Poisson distributed inputs but they equally apply to other members. Given the homogeneity assumption for the input we find the log-partition to be 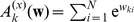 [33].

The prediction model has to reflect the dynamics of the state 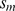. At each time point 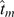 the spiking output projects the 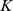-dimensional state 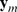 to the discrete value 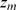, which is then projected in the next step to 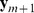. Using the independence properties, that emerge from these dynamics, the prediction model factorizes to
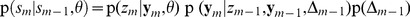
(14)The last term determines the distribution over the inter-spike intervals 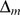. Assuming Poisson distributed spike times 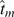 this is given by an exponential distribution with mean 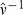, i.e.
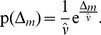
(15)The second part of (14) deterministically updates the EPSPs. Using the simple kernel function (12) the lateral EPSPs can be updated online
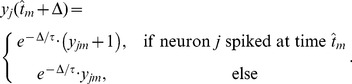
(16)Since this is a deterministic function the probability distribution 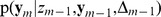 in (14) collapses to a single mass point, where the update equation (16) is fulfilled. The second and third parts of (14) project the spiking network output to the 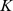-dimensional space of the EPSP time courses (2). The first part of the prediction model projects it back to a discrete variable drawn from a multinomial distribution 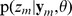. Using Bayes rule, we can decompose this into
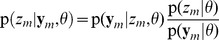
(17)The likelihood term can again be expressed in terms of an exponential family distribution
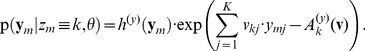
(18)For each neuron 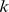 the prior probability to fire is determined by the excitability parameter, i.e. 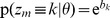. In [32] a learning rule was presented for these network parameters, which equally applies to the framework presented here. For simplicity however, we can assume that all neurons have the same prior probability to fire, i.e. 
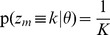
.

Under the homogeneity condition and if the synaptic weights obey 
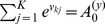
, the log-partition function 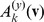 becomes constant over 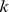. It has been shown that this condition emerges automatically from the STDP rules (7) [33]. Using this, the probability of generating a state sequence 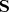 using forward sampling can be directly linked to the network dynamics. The true posterior distribution (8) and the proposal distribution for forward sampling only differ in the normalization. Forward sampling is done, by explicitly normalizing the state update in (4) at each time point 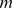 [20]. This normalization is given by 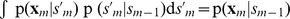, from which we find the proposal distribution to be given by
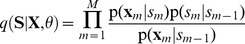
(19)

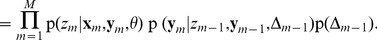
(20)This recovers the result of equation (5). The first term of the second line can be written using (13), (17) and (18)
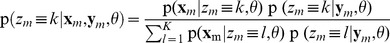
(21)

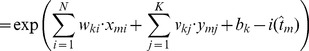
(22)with 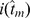 given by (3). Here we have used that the marginal in the denominator of (17) does not depend on 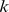 and so do 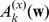 and 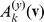 under the conditions described above. Therefore they cancel out through the normalization (3). Comparing this result with the neuron dynamics (6) and (5) it is easy to verify that the WTA circuit correctly realizes the HMM forward sampler (19).

#### Details to *STDP installs a stochastic approximation to EM parameter learning*

In this section we derive the optimal updates for the model parameters in terms of the expectation-maximization (EM)-algorithm and show that the STDP rules (7) are stochastic approximations. The goal of the EM optimization is to minimize the error between the model likelihood 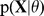 and the empirical distribution over input sequences, which we denote by 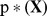. A natural way to express this error is the Kullback-Leibler divergence. Thus, the update can be derived by minimizing
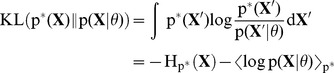
(23)where 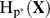 is the entropy of the true input distribution and 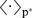 denotes the expectation with respect to 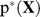. We are interested in a solution to 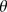 that minimizes (23). Since 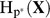 is constant for a given input sequence 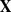, it can be ignored and minimizing (23) becomes equivalent to maximizing the expected log-likelihood 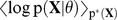. The derivative of the log-likelihood can be simplified to
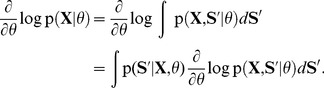
(24)The integral can again be written in terms of an expectation. The condition for the maximum likelihood becomes
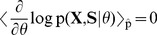
(25)where 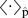 denotes the expectation with respect to 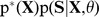. The derivative in this last form can be easily calculated. By inserting the model joint distribution (4) it yields for the model parameters 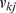 of neuron 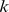


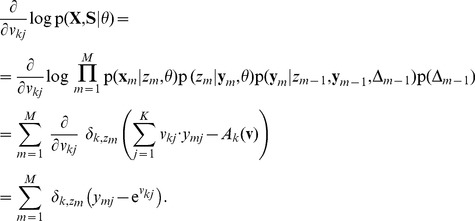
(26)A similar result can be found for 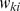. Here, 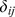 is the Kronecker delta, which is one if 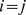 and zero otherwise. Inserting this result into (25), setting the derivative to zero and rearranging the terms, we identify the optimal model parameters
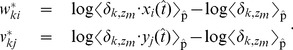
(27)In the E-step the expectations 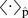 are evaluated. Note that the expectations are taken over the whole sequence of 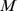 output spikes. In the M-step the parameters are updated to their new values. An estimate of these expectations is computed by the network generating output spike sequences. A local minimum of (23) can be found by iteratively evaluating the E- and M-step.

We will now show that the STDP protocol introduced here converges stochastically to the same result as the EM updates (27). We will derive this results for the lateral weights 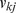 only, since adaption for other parameters is straightforward. Including the reward mechanism, the weight update consists of two stochastic processes: the forward sampling and the stochastic decision for the rejection step (11). The updates are made for each output spike of the network and therefore they will always fluctuate. In our analysis we are interested in the equilibrium point of these fluctuations for some given target distribution 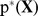. This can be expressed for the expected weight update using (7),(9) and (11), for which we get
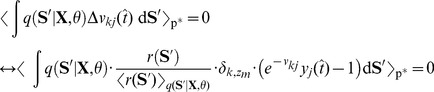
(28)which by inserting equation (10) yields
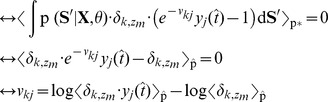
which is equivalent to the solution of the EM-algorithm (27).

#### Details to *A refined EM approximation using rejection sampling*

Here we present additional details to the rejection sampling algorithm that was used throughout the numerical experiments. The algorithm requires to evaluate two quantities that evolve on different time scales. The synaptic weight updates need to be updated on each spike, whereas the importance weights (10) need to be tracked over a whole input sequence.

The importance weight over sequence 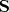 is given by (10) which can be verified by inserting equation (19) and (4) into (10). Using (3) we find that this quantity computes to
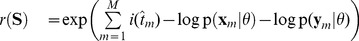
(29)where the marginals 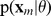 and 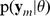 are given by
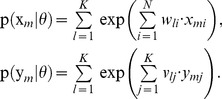
(30)The term 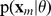 is arbitrary since it cancels out in the rejection sampling algorithm, but we found that (30) achieves better performance including the dependence on 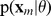, when using the the rejection sampler with the simple tracking mechanism for 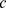. The performance of the rejection sampling algorithm essentially depends on the variance of the importance weights. The lower this variance is, the more generated sequences will be accepted. Since the importance weights are only needed to compare the quality of different proposed hidden state trajectories, all fluctuations that depend on the feedforward weights and inputs only, can be discarded. Explicitly subtracting 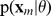 allows to minimize the fluctuations injected by the feedforward synapses. This modification had a large impact on reducing the number of rejected trajectories and therefore increased learning speed.

The likelihood of an input sequence 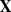 can be approximated using a set of 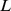 paths 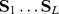 sampled from (5) given by
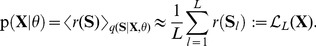
(31)In the simplest approximation the expectation can be taken over a single path. Thus, we find that the sequence log-likelihood can be directly approximated by (29), i.e.
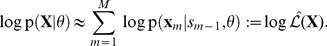
(32)In the AGL experiments this simple approximate likelihood was used to distinguish between grammatical and non-grammatical sequences.

The probability of generating a sequence 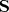 through the state space is given by the proposal distribution 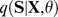, which was defined in equation (5). The bias between this and the model distribution 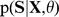, is given by the importance weight 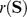, which we have derived earlier (10). This bias can be eliminated using rejection sampling, i.e. accepting the sampled sequences based on a stochastic decision proportional to the importance weight. In the neural network we implemented this using an eligibility trace of synaptic weight changes [47]. The weight updates were accumulated over the whole input sequence
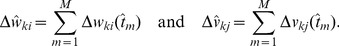
(33)The synaptic weights can be learned by modulating the learning rate 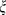 when incorporating the synaptic weight changes (33) at the end of a sequence. The learning rate must be modulated according to the importance weights. In the simulations we used a stochastic binary decision, whether to accept or reject the sampled sequence
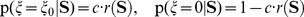
(34)where, 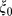 is a constant learning rate and 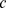 is a constant that scales the average number of rejected samples. The probability of accepting a path 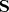 is directly proportional to the importance weights. Using this, we immediately find that the mean number of rejected samples 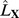 for an input sequence 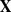 is inversely proportional to the sequence likelihood, i.e.
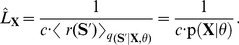
(35)The average learning rate assigned to a sampled sequence 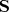 depends on the probability of sampling 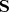 from the proposal distribution and the number of times the sequence is resampled. Using this, (35) and (34) we find the expected learning rate associated with a state sequence 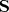 to be given by
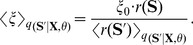
(36)


The constant 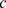 in (34) can be used to control the average number of rejected samples. We used a simple linear tracking algorithm for 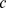 in the logarithmic domain. Whenever a path was accepted 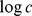 was decreased by 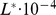, if the path was rejected 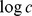 was increased by 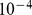. As learning proceeds the network converges to an equilibrium acceptance rate, determined by 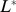. Throughout the experiments this parameter was tuned to achieve the desired mean number of samples over the whole training session. A quantitative comparison between the learning performances achieved with the batch algorithm and this tracking mechanism, is given in Fig. 8.

In the batch version of the algorithm a set of sampled paths with a fixed size 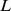 was used to compute 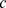 directly, which was chosen such that the distribution over the 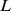 paths was correctly normalized. Using (34) we find this to be fulfilled for
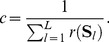
(37)A sequence 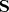 was then chosen at random from the set of 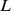 sampled sequences. The importance sampler was realized by directly weighting the synaptic changes by the scalar value of the normalized importance weight, i.e. 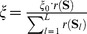.

### Simulations and data analysis

All simulations were done in Matlab (Mathworks), directly implementing the derived equations without discrete time approximations. The population output rate 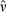 was tuned to give an average output rate of 5–20 Hz per neuron. Prior to learning all weights were set to small equally distributed random values. The weight updates were incorporated using a constant learning rate 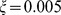.

Other than in the theoretical analysis where synaptic delays were neglected for the sake of simplicity, we used synaptic delays of 5 ms for the lateral excitatory synapses in the numerical experiments. We also used a more realistic double exponential EPSP kernel of the form [28]

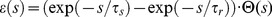
(38)where 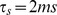 and 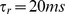 are the time constants of the falling and rising edges of the EPSP kernel, respectively. The above theoretical analysis applies equally to this kernel, but would be slightly more complex since each of the two exponential decay terms comprises a piece of memory which has to be reflected in the network state 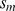.

The diagonal of the weight matrix 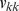 was set to zero and these weights were excluded from learning. Instead, a refractory mechanism was used with a kernel given by [28]

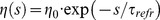
(39)where 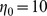 is the maximum amplitude of the refractory kernel, 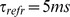 is the refractory time constant and 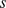 is the time elapsed since the last output spike. Equation (39) was subtracted from the membrane potential (1).

#### Details to *Learning to predict spike sequences through STDP*

The input patterns were generated by drawing for each afferent neuron and each pattern a value from the Beta distribution with parameters 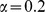, 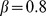 and multiplying this value with the maximum rate of 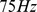. Using these rate patterns, input spikes were then generated by creating independent spike events from a Poisson process.

To facilitate the interpretability of the network output, we applied a smoothing and sorting algorithm. The spike statistics were estimated using the perievent time histogram (PETH) on the network output [6]. The network output rates were computed for time bins of 1 ms and then filtered with a Gaussian filter function (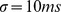) to give the smoothed single-trial estimated rates 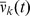. These spike histograms were averaged over 100 trial runs to give the time estimated rates 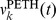 for each neuron 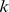. For neuron sorting we evaluated the point in time with the highest activity
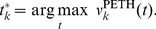
(40)This was used as criterion to determine the rank index of the output neurons for sorting. The PETHs for all sequences of a learning problem were concatenated before evaluating the maximum firing time (40) to ensure a visual separation between neurons that fired preferentially during one specific sequence. This neuron order was also used to sort the rows and columns of the synaptic weight matrix shown in Fig. 2 (neurons that fired on average less than one spike per sequence were excluded from this plot).

To quantify the similarity between spontaneous and evoked network activity we used Spearman's rank correlation [6]. The correlations were computed by evaluating the rank correlation between the PETH computed on a single spontaneous sequence and the evoked activity averaged over 100 trial runs. For the evaluation only the neurons that produced at least one spike during the spontaneous run were used. The firing rates in Fig. 4A,B were estimated over 100 input sequences. Only the time window during which pattern *a* was present on the input was analyzed. Neurons that fired with average rates less than 10 Hz during these time windows were excluded from the analysis. Neurons with rates above 10 Hz for patterns appearing in one sequence, but not the other, were classified as context specific. Those that fired rates above 10 Hz during both sequences were classified as context unspecific.

#### Details to *Mixed selectivity emerges in multiple interconnected WTA circuits*

Here a linear classifier was used to identify separating planes in the network activity. We trained a soft-margin support vector machine with linear kernels [19], [72], [73] to classify the network activity during the delay phase of sequence *AB-delay-ab* against that of *BA-delay-ba*. The resulting linear models were used to classify 50 test samples from each of the two sequences. Sequences that were at any point in time on the wrong side of the separating plane were reported as wrongly classified. The mean classification rates over these test samples were reported.

To illustrate the network state during the holding phase we used the dynamic PCA (jPCA) method in experiment 2. This method was recently introduced as an extension to normal PCA, with better applicability to dynamical data [39]. We applied this method on the smoothed network activities 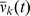 of all network neurons. The jPCA identifies the plane that is aligned with the fastest rotation in the data set. Briefly, the jPCA first uses a preprocessing step in which normal PCA is performed on the data to reduce the dimensionality. We used the first 6 PCA components as suggested in [39]. Subsequently a projection from the neural state to its slope is found. A skew-symmetric matrix is constructed that projects the PCA components into its first order derivatives. The solution to this constraint optimization problem is a matrix defining the best-fitting rotational linear dynamical system which can describe the data set (see the supplementary derivation of [39] for details). The orthogonal basis of the jPCA is then given by the real plane associated with the eigenvectors with largest imaginary eigenvalues of this projection matrix. This plane is aligned with the fastest rotation in the data set.

#### Details to *Trajectories in network assemblies emerge for stationary input patterns*

In this experiment we employed homeostatic mechanism to control the excitabilities 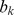. A detailed derivation of this intrinsic plasticity was presented in [44]. Following this approach we slowly regulated 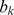 over time to maximizes the entropy of the network output by demanding that the overall output rate of each neuron, measured over a long time window 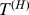, converges to the target rate 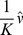, i.e. 
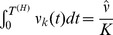
 for each neuron 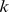. A stochastic approximation to that can be achieved by updating the excitabilities 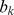 in (1)
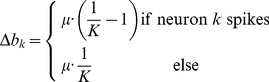
(41)where 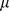 is an update rate we have chosen to be 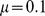 in this experiment. This mechanism assures that all network neurons participate on average equally in the representation of the hidden state. If 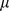 is chosen small enough this method assures that all network neurons participate equally in the representation of the hidden state [44].

#### Details to *Learning the temporal structure of an artificial grammar model*

Here we used two data sets from [27] - the 12 sequences reported there in appendix A for training and the 20 sequences from table 1 for testing. In each training iteration we randomly drew one example input sequence from the train set. For testing we created 100 legal and illegal sequences randomly drawn from the test set. The sequences were encoded using sparse input patterns, encoded by 10 input neurons, two of which fired with a rate of 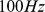 for 50 ms for each of the five input pattern, while the others remained silent. All spike patterns were not kept fixed but generated newly at each occurrence of the pattern and also during replay for rejection sampling. In the AGL experiments, the initial network state was reset to zero 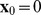, 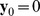 before a new input sequence was presented. To classify grammatical against non-grammatical sequences the one-sample approximation of the log-likelihood (32) was computed for all test sequences. A threshold was computed by taking the mean of these log-likelihoods. Sequences 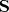 for which 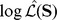 lied above this threshold were classified as grammatical, all others as non-grammatical.

#### Details to *Comparison of the convergence speed and performance of the approximate algorithms*

In this experiment, random teacher HMMs were generated by drawing initial state, observation and transition probability tables from a Beta distribution with 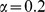 and 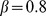 and then normalizing the tables to proper conditional probabilities. The models had 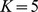 states and 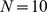 discrete observations. These models were then used to generate observation sequences. We drew a training set of 200 and a validation set of 2000 sequences of length 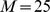. The complete training data set was repeatedly present to the network. We refer to the presentation of the whole batch of training sequences as an epoch. The weight updates for the WTA circuit were applied at the end of each training sequence. In each epoch all sequences were presented in random order. For the Baum-Welch algorithm (which is not an online algorithm) the updates were computed over all sequences in each epoch (batch learning). We generated 50 trials using 50 different teacher HMMs. The performance of rejection sampling was assessed for the two algorithms to evaluate the normalizing constant 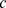 – the exact version (37) and the linear tracking algorithm.
